# Revolutionizing Cancer Treatments through Stem Cell-Derived CAR T Cells for Immunotherapy: Opening New Horizons for the Future of Oncology

**DOI:** 10.3390/cells13181516

**Published:** 2024-09-10

**Authors:** Hemant K. Mishra, Alex Kalyuzhny

**Affiliations:** 1 Cellinfinitybio, San Francisco, CA 94107, USA; 2 Biotechne 2, Minneapolis, MN 55413, USA; kalyu001@umn.edu

**Keywords:** induced pluripotent stem cells (iPSCs), chimeric antigen receptor (CAR), immunotherapy, differentiation

## Abstract

Recent advances in cellular therapies have paved the way for innovative treatments of various cancers and autoimmune disorders. Induced pluripotent stem cells (iPSCs) represent a remarkable breakthrough, offering the potential to generate patient-specific cell types for personalized as well as allogeneic therapies. This review explores the application of iPSC-derived chimeric antigen receptor (CAR) T cells, a cutting-edge approach in allogeneic cancer immunotherapies. CAR T cells are genetically engineered immune cells designed to target specific tumor antigens, and their integration with iPSC technology holds immense promise for enhancing the efficacy, safety, and scalability of cellular therapies. This review begins by elucidating the principles behind iPSC generation and differentiation into T cells, highlighting the advantage of iPSCs in providing a uniform, inexhaustible source of CAR T cells. Additionally, we discuss the genetic modification of iPSC-derived T cells to express various CARs, emphasizing the precision and flexibility this affords in designing customized therapies for a diverse range of malignancies. Notably, iPSC-derived CAR T cells demonstrate a superior proliferative capacity, persistence, and anti-tumor activity compared to their conventionally derived counterparts, offering a potential solution to challenges associated with conventional CAR T cell therapies. In conclusion, iPSC-derived CAR T cells represent a groundbreaking advancement in cellular therapies, demonstrating unparalleled potential in revolutionizing the landscape of immunotherapies. As this technology continues to evolve, it holds the promise of providing safer, more effective, and widely accessible treatment options for patients battling cancer and other immune-related disorders. This review aims to shed light on the transformative potential of iPSC-derived CAR T cells and inspire further research and development in this dynamic field.

## 1. Introduction

The application of induced pluripotent stem cells (iPSCs) represents a groundbreaking advancement in regenerative medicine and personalized therapeutics [1]. iPSCs, reprogrammed from adult somatic cells, possess the unique ability to differentiate into any cell type within the human body, holding immense potential for a wide array of medical applications [2]. This transformative technology has opened avenues for disease modeling, drug discovery, tissue engineering, and cellular replacement therapies, offering a promising frontier in the quest for innovative and patient-tailored medical interventions [3]. Up to this point, utilizing autologous T cells for manufacturing chimeric antigen receptor T (CAR T) products has been the standard practice [4]. However, this method has several drawbacks, including the production time, cost, manufacturing delays, and reliance on the functional fitness of the patient’s T cells, which is often compromised by disease state or prior treatment(s) [5]. Moreover, autologous therapies are challenging to scale and exhibit non-uniformity across the intervention group, making it difficult to assess their efficacy due to numerous variables that are hard to control [6].The integration of iPSCs into the domain of CAR T-cell therapy signifies a paradigm shift in the field of immunotherapies [7]. iPSCs, with their remarkable capacity to differentiate into various cell types, including T cells, offer a renewable and customizable source for CAR T-cell production. This innovative approach not only addresses the challenges associated with traditional CAR T-cell therapies but also paves the way for personalized, scalable, invariable, and precisely engineered cellular immunotherapies [2,7,8]. Despite revolutionizing hematologic malignancy treatments, immunotherapy has thus far proven ineffective against solid tumors [9]. Unlike liquid tumors, solid tumors often exhibit immunosuppressive microenvironments that impede immune cell infiltration and activation [9]. Additionally, tumor heterogeneity and the presence of self-antigens complicate effective immunotherapeutic targeting. Overcoming these obstacles may require multiple modifications, which are challenging to achieve with autologous cells. Stem cells could offer a better solution for creating effective CAR T cells with multiple endo-modifications and knocked-out genes, which may be required to combat a hostile tumor microenvironment, resulting in longer survivability and greater overall efficacy.

## 2. Generation of Allogeneic CAR T Cells

Allogeneic CAR T cells can be produced by cells harvested from the same patient (autologous). However, there are many disadvantages of this, including the cost of production, duration, and poor quality. Leukapheresis collection is the primary and most utilized starting material for autologous CAR T-cell manufacturing [10,11,12]. Conversely, in the allogeneic setting, apheresis is performed on healthy adult volunteers [13]. Using healthy donors provides a high number of cells from a single volunteer, and their peripheral blood mononuclear cells are fit, since they do not undergo chemo- or radiotherapy, unlike cancer patients [14].

## 3. Cord Blood-Derived CAR T Cells

Hematopoietic stem cells (HSCs) can be isolated from cord blood from the umbilical cord and placenta after childbirth and can differentiate into various blood cell types, including T cells. HSCs from cord blood are induced to differentiate into T cells using specific cytokines and growth factors. These T cells are expanded in vitro to achieve the necessary cell numbers for therapy. T cells are genetically modified to express chimeric antigen receptors (CARs) [15].

In addition to HSCs, umbilical cord blood (UCB)-derived T cells can also be used to develop CAR T cells. UCB-derived T cells have a naive phenotype, which may lead to a more robust and long-lasting immune response and can also be considered for allogeneic CAR T-cell development [15,16]. In the context of hematopoietic stem cell transplantation (HSCT) for treating hematological malignancies, UCB transplantation has shown better results than matched unrelated donors and similar outcomes compared to matched related donor transplantation in terms of GVHD incidence, late complications, and overall survival [17]. CAR T cells derived from UCB have already demonstrated feasibility and efficacy, as UCB-derived CAR T cells can recognize and kill target cells [18]. One of the disadvantages of using cord blood cells, however, is the variability in the quality and quantity of T cells from different cord blood samples [18,19,20].

## 4. iPSC-Derived CAR T Cells

**Figure 1:**
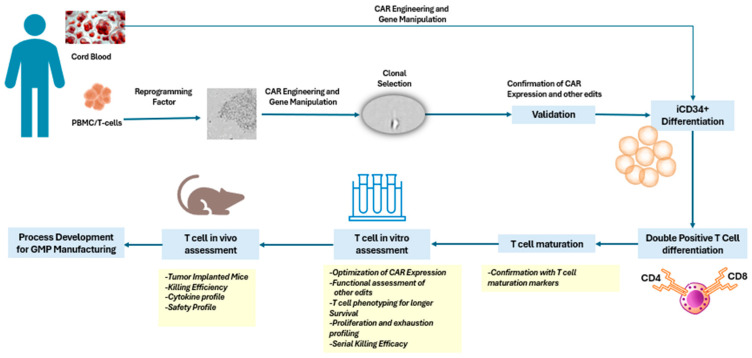
Flow chart of producing allogeneic CAR T cells from cord blood or iPSCs.

The regeneration of antigen-specific T cells via iPSCs was first reported in 2013 and represents a potential source of cytotoxic T lymphocytes (CTLs) [2]. This strategy involves reprogramming T cells into iPSCs and then differentiating them back into T cells with rejuvenated phenotypes. iPSCs are generated by reprogramming adult somatic cells (e.g., skin fibroblasts, PBMCs, or T cells) using specific transcription factors (e.g., Oct4, Sox2, Klf4, and c-Myc) [2,21]. These types of stem cells can differentiate into any cell type, including T cells [2]. This process involves a series of culture conditions and cytokines that mimic natural T-cell development (Figure 1). During or after differentiation, iPSC-derived T cells are transduced with CAR constructs using viral or non-viral methods [22]. However, previous reports have shown that these cells can exhibit unexpected phenotypes. In particular, cytotoxic T cells derived from second-generation CD19 CAR-transduced iPSCs were reported to have the properties of γδT cells, partially expressing CD8αα but not CD8αβ, according to gene expression profiles and CAR-independent cytotoxicity [2,8,23]. Recent reports have indicated improved differentiation protocols to synthesize CD8αβ-expressing T cells that exhibit effector functions more closely resembling primary T cells [8]. To derive CD8αβ iPSC T cells from CAR-modified iPSCs, the selection of optimal CAR constructs that avoided tonic signaling during CD8αβ T-cell differentiation should be considered [8]. Studies have reported that enhanced the CD3ζ-mediated signaling pathway can be inhibited by downmodulating the intracellular immunological checkpoint molecules DGKα and DGKζ [8,24].

CAR transduction in these iPSC T cells has been confirmed to work as effectively as in primary T cells on a B-cell malignancy animal model when supported with an IL-15-mediated third signal [25]. However, unlike hematological malignancies, solid tumors are more resistant to cellular immunotherapies due to challenges in accessibility and sustained T-cell effector function at the local tumor site [26]. iPSC-derived CAR T cells have a greater advantage over autologous-derived CAR T cells against solid tumors because they can facilitate multiple editing with greater efficiency, uniformity in CAR T-cell production, and unlimited supply [27,28]. The proliferation and persistence of CAR T cells can be facilitated by IL-15/IL-15RA engineering [8]. Additionally, iPSC-derived T cells can be genetically edited to enhance their functionality, persistence, and safety [29]. iPSC-derived CAR T cells have potential for creating invariable “off-the-shelf” CAR T-cell products that are ready for immediate use [27,28,29]. Some of the challenges of using iPSCs are that differentiation protocols must be optimized to ensure efficient and consistent production of functional T cells [28].

In both cases, the overall structure of the CAR T cell involves the integration of the CAR construct, which provides the specificity and activation signals necessary for targeting and killing cancer cells [30,31]. The choice of source—cord blood or iPSCs—affects the availability, scalability, and potential clinical applications of these allogeneic CAR T-cell therapies [32]. Some of the FDA-approved CAR T-cell therapies are mentioned in Table 1.

In addition to this, a number of phase 1 clinical trials have demonstrated promising results in allogeneic CAR T-cell therapies. For example, in two multicentric phase 1 trials (NCT02808442 and NCT02746952), allogeneic anti-CD19 CAR T cells (UCART19) demonstrated their feasibility with encouraging results [41]. In a separate clinical trial, UCART19 CAR T cells demonstrated their safety and antileukemic activity to treat patients with relapsed B-cell acute lymphoblastic leukemia [42]. In a single-arm phase 2 study (NCT04340154), having sequential CD19 and CD22 CAR T cells for childhood refractory or relapsed B-cell acute lymphocytic leukemia demonstrated deep and sustained responses [43]. In another phase 1b clinical trial, CD22 CAR T demonstrated safety and high response rates in pediatric and adult B-ALL [44]. In a study using CRISPR/Cas9 engineering for universal dual CAR (CD19/CD22), the authors demonstrated manageable safety profile and prominent antileukemic activities in relapsed/refractory B-cell acute lymphoblastic leukemia [45].

## 5. CAR T-Cell Structure and Design

**Figure 2:**
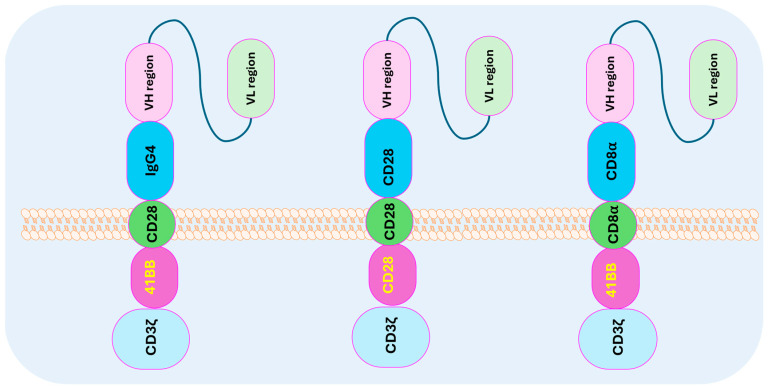
Key components of CAR T-cell design and generation: CD3ς (cluster of differentiation), a component of T-cell co-receptors involved in activation; 41BB (CD137), a co-stimulatory receptor for persistence and survival; CD28, which co-stimulates CAR T cells for a rapid anti-tumor response; CD8α, which decreases activation-induced cell death; IgG4, which improves CAR T-cell targeting.

The structure of a CAR T cell is built around a synthetic receptor that is introduced into T cells to confer specificity for tumor antigens. The design of the CAR involves several key components:

A typical CAR T cell has an extracellular antigen-binding domain (Figure 2), which is typically derived from the variable regions of a monoclonal antibody; this domain is responsible for recognizing and binding to a specific antigen on the surface of cancer cells. Just below the antigen-binding domain rests the hinge region, which is a flexible linker connecting the antigen-binding domain to the transmembrane domain, allowing the CAR to reach and interact with the target antigen effectively. The transmembrane domain anchors the CAR to the T-cell membrane and ensures the stability and or the flexibility of the receptor. In addition to this, the intracellular signaling domains contains activation signaling domains like CD3ζ, which are essential for T-cell activation, and one or more co-stimulatory domains (such as CD28 or 4-1BB) that enhance the T cell’s proliferation, survival, and cytotoxic activity. The optimization of specific CAR T cells for a particular purpose can be achieved by experimenting with various permutations and combinations of these components, as detailed in Table 2.

The overall design of CAR T cells enables them to recognize and kill cancer cells in an antigen-specific manner, independent of major histocompatibility complex (MHC) presentation. This makes CAR T-cell therapy a powerful and versatile approach for treating various types of cancer, especially hematologic malignancies. Advances in CAR design continue to improve their efficacy, safety, and applicability, including the use of allogeneic sources like cord blood and induced pluripotent stem cells (iPSCs) to create “off-the-shelf” CAR T-cell products.

## 6. Selection of Optimized scFv

**Figure 3:**
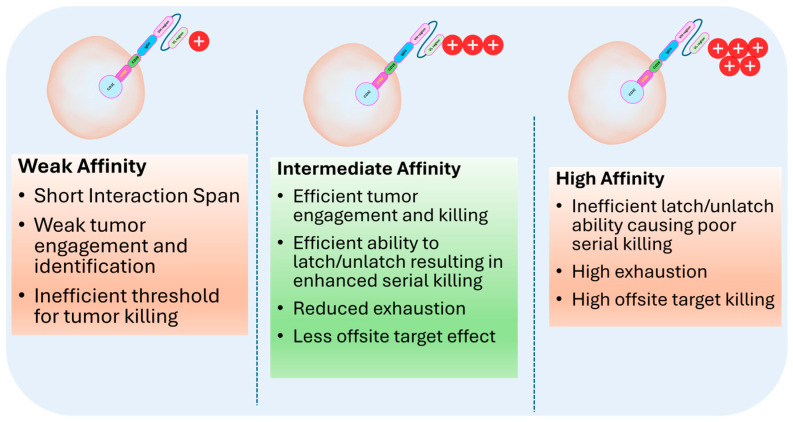
Illustration of Goldilock zone to identify optimal scFvs best suited for CAR T-cell efficacy and safety.

The selection of single-chain variable fragment (scFv) candidates for a particular CAR T cell involves identifying the antibody fragments that exhibit optimal specificity and affinity for the target antigen expressed on cancer cells. This process typically begins with screening a diverse library of antibody sequences to isolate those that bind effectively to the desired antigen. Once potential scFv candidates are identified, they undergo rigorous testing to assess their binding kinetics, stability, and ability to elicit a robust immune response when integrated into the CAR structure. Further optimization may involve engineering the scFv to enhance its binding properties and minimize off-target effects. The ultimate goal is to select an scFv that not only targets the cancer cells with high precision but also contributes to the overall efficacy and safety of the CAR T-cell therapy. Moderate-affinity scFvs are often more optimal for CAR T-cell design because they can effectively engage and disengage from the target antigen. This enhances the CAR T cells’ serial killing ability and reduces off-target effects. (Figure 3).

## 7. Enhancing the Allogenicity and Efficacy of CAR T Cells

**Figure 4:**
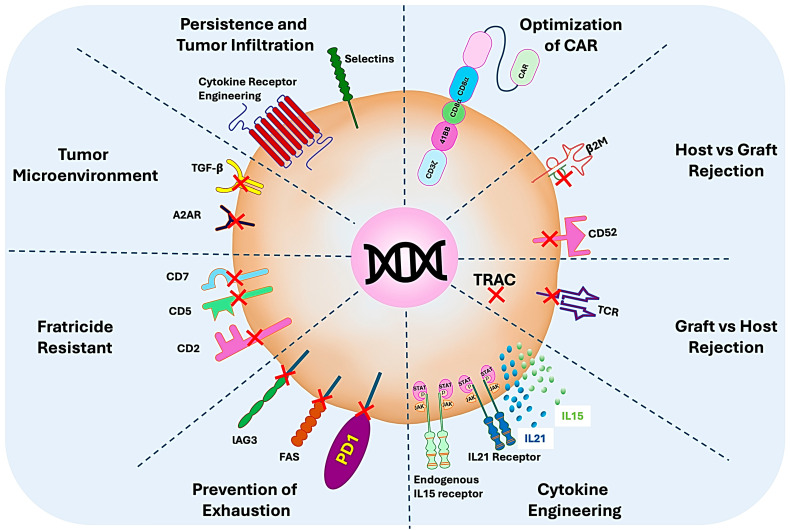
Illustration of various edits to make CAR T cells allogeneic and resistant to hostile tumor microenvironment.

Several genetic modifications can be considered to make CAR T cells allogeneic and resistant to the hostile tumor microenvironment; some of these modifications are suggested in Figure 4. The production of allogeneic CAR T cells involves engineering T cells from healthy donors to be universally applicable rather than patient specific. A critical step in this process is knocking out the T-cell receptor (TCR) alpha constant (TRAC) gene using techniques such as CRISPR/Cas9. This knockout prevents the T cells from recognizing and attacking the recipient’s healthy tissues, thereby reducing the risk of graft-versus-host disease (GVHD) [46,47]. The modified T cells are then transduced with a chimeric antigen receptor (CAR) construct, enabling them to target specific cancer antigens. This approach offers the advantages of scalable production, immediate availability, and potentially lower costs compared to autologous CAR T-cell therapies.

To mitigate host-versus-graft rejection in allogeneic CAR T-cell therapies, genetic modifications such as knocking out Beta-2 microglobulin (β2M) or CD52 can be employed [47,48]. β2M knockout disrupts the expression of major histocompatibility complex class I (MHC I) molecules on the surface of CAR T cells, preventing their recognition and destruction by the recipient’s immune system. Similarly, CD52 knockout removes the target for the monoclonal antibody alemtuzumab, which is used to deplete the recipient’s immune cells, thereby protecting the infused CAR T cells from being attacked [47]. These modifications enhance the persistence and efficacy of allogeneic CAR T cells, making them more viable for widespread clinical use.

To enhance the survival of CAR T cells, cytokine receptor engineering, particularly involving IL-15 and IL-21, plays a crucial role in enhancing the efficacy and persistence of CAR T cells. IL-21 receptor engineering can promote the proliferation, persistence, and functionality of CAR T cells, boosting their anti-tumor activity [49]. IL-15 receptor engineering similarly enhances CAR T-cell survival and proliferation. By introducing membrane-bound IL-15 or its receptor complex (IL-15/IL-15RA) into CAR T cells, researchers can provide continuous autocrine stimulation, reducing the need for external cytokine support [50]. These modifications not only improve the CAR T cells’ ability to persist and function in the hostile tumor microenvironment but also reduce their dependency on the host’s cytokine milieu, leading to more robust and durable therapeutic responses.

The hostile tumor microenvironment promotes CAR T-cell exhaustion, which is very critical for maintaining the efficacy of CAR T-cell therapies. T-cell exhaustion occurs due to chronic antigen exposure and is characterized by the upregulation of inhibitory receptors such as PD-1, LAG-3, and Fas, which impair T-cell function. To combat this, genetic modifications can be made to CAR T cells to either knock out or inhibit these receptors [51,52,53]. PD-1 knockout or blockade can prevent T-cell inhibition, enhancing their persistence and cytotoxicity [51]. Similarly, targeting LAG-3, another inhibitory receptor, can further sustain T-cell activity and prevent exhaustion [52]. Additionally, manipulating the Fas pathway, which mediates activation-induced cell death, can reduce T-cell apoptosis and prolong their survival [53]. These strategies collectively help maintain the functional state of CAR T cells, enhancing their long-term anti-tumor efficacy.

Fratricide resistance in CAR T cells is crucial for therapies targeting T-cell antigens such as CD2, CD5, and CD7. Fratricide occurs when CAR T cells attack each other due to the expression of these target antigens on their own surface. To prevent this, genetic modifications can be employed. Knocking out CD2, CD5, or CD7 in CAR T cells can eliminate the target antigen, preventing self-recognition and fratricidal killing [54,55,56]. This ensures that the engineered CAR T cells can survive, proliferate, and maintain their anti-tumor activity without being compromised by mutual destruction. Such modifications enhance the effectiveness and longevity of CAR T-cell therapies targeting T-cell malignancies.

In addition to fratricide, immunosuppression is very common in tumor microenvironment. Transforming growth factor-beta (TGF-β), which is often upregulated in tumors and contributes to immune evasion and suppression, plays a key role in this. TGF-β can inhibit the proliferation and function of CAR T cells, reducing their effectiveness. It suppresses the activation and cytokine production of these cells. Furthermore, TGF-β also promotes the differentiation of regulatory T cells (Tregs), which further contributes to an immunosuppressive environment. The presence of TGF-β in the tumor microenvironment can directly impact the efficacy of CAR T-cell therapy by diminishing their anti-tumor responses [57].

Another key molecule that has an immunosuppressive effect on CAR T cells in the tumor microenvironment is adenosine. Adenosine, a molecule often elevated in the tumor microenvironment, binds to A2A adenosine receptors on immune cells. This interaction has several implications in CAR T-cell therapy. The activation of A2A receptors by adenosine can suppress T-cell activation and function. It impairs CAR T-cell cytotoxicity and reduces their ability to kill tumor cells by impacting their long-term persistence and efficacy. The immunosuppressive effects of adenosine on T cells can hinder the expansion and persistence of CAR T cells in the tumor microenvironment [58].

Overall, both TGF-β and A2A adenosine receptors play crucial roles in shaping the immune landscape within tumors and can significantly influence the success of CAR T-cell therapies. Strategies to counteract their effects, such as combining CAR T cells with TGF-beta inhibitors or A2A receptor antagonists, are actively being researched to enhance the efficacy of these treatments. CAR T cells can also be engineered with various cytokine receptors, which can facilitate their infiltration and efficacy specially in solid tumors [59]. Another approach to combat CAR T-cell exhaustion is metabolic rewiring, which can be achieved by enhancing mitochondrial metabolism [60], the inhibition of CD38 enzymatic activity [61], and inosine or stemness induction [62,63].

There are several potential risk factors of using CAR T cells, including insertional mutagenesis during CAR engineering, variabilities across the patient group, cytokine release syndrome, tumor lysis syndrome, etc. To address some of these issues, the FDA has issued a set of updated guidelines regarding these considerations during the development of CAR T-cell products [64]. Some of the key features are mentioned in Table 3.

For iPSC-derived CAR T cells, it is crucial to carefully analyze the release product for purity as well as the absence of iPSCs to avoid any potential teratoma formation upon injection.

## 8. Future Direction and Scope

The scope and prospects of stem cell-derived CAR T cells hold great promises and potential for revolutionizing cell-based therapies against various cancers and other autoimmune disorders. Several preclinical studies have demonstrated various advantages of iPSC-derived CAR T-cell therapies. Iriguchi, S., et al. showed the consistency and scalability of iPSC-derived CAR T cells [65]. Kawai, Y., et al. showed that highly proliferative cytotoxic T cells can be generated by pluripotency reprogramming [66]. Epigenetic remodeling in iPSC-derived T cells also led to enhanced anti-tumor activity [22]. Itoh, M., et al. demonstrated the possibility of generating antigen-specific CAR T cells for melanoma using iPSC technology [67]. Harada, S., et al. generated dual-antigen receptor T cells from iPSCs and demonstrated clear survival and a robust tumor suppressive effect [68].

As the advancement of gene editing by various approaches continues along with stem cell technologies, stem cell-derived CAR T cells are poised to offer personalized, off-the-shelf, and uniform treatments that address the limitations of conventional CAR T-cell therapies. The stem cells, specifically iPSCs, could facilitate the capabilities to generate an abundant supply of universal donor T cells, which can not only reduce the cost but also broaden the accessibility while maintaining the uniformity of the product. The iPSCs could also facilitate multilayered gene editing without compromising the DNA stability, which can be ensured by clonal selection. These features can enhance the safety and efficacy of CAR T cells by improving the targeting precision and minimizing the risk of adverse or offsite target effects. By considering these strategies, stem cell-derived CAR T cells could potentially offer better, effective, and adaptable solutions for a wide range of cancers and autoimmune disorders.

## 9. Conclusions

Induced pluripotent stem cells (iPSCs) hold remarkable potential in advancing CAR T-cell therapies, offering several distinct advantages that could revolutionize the field. iPSCs provide a versatile and renewable source of T cells, enabling the development of uniformly engineered CAR T cells with consistent quality. Their capacity for multiple genetic modifications allows for sophisticated enhancements, such as overcoming tumor escape mechanisms and mitigating immune suppression. Additionally, iPSCs can be derived from a range of donors, potentially overcoming issues of allogeneic rejection and expanding the availability of CAR T-cell therapies. By leveraging iPSCs, researchers can create highly effective, customizable, and broadly applicable CAR T-cell therapies, addressing current limitations and paving the way for more personalized and scalable cancer treatments.

## Figures and Tables

**Table 1:** Some of the prominent FDA-approved CAR T-cell therapies.

CAR T-Cell Therapy Name	Target Antigen	Indication	Manufacturer	Reference
Kymriah (tisagenlecleucel)	CD19	B-cell acute lymphoblastic leukemia (ALL)	Novartis	FDA approval (2017) [33,34]
Yescarta (axicabtagene ciloleucel)	CD19	Large B-cell lymphoma	Kite Pharma/Gilead	FDA approval (2017) [35]
Kymriah (tisagenlecleucel)	CD19	Large B-cell lymphoma	Novartis	FDA approval (2018) [34]
Yescarta (axicabtagene ciloleucel)	CD19	Follicular lymphoma	Kite Pharma/Gilead	FDA approval (2021) [36]
Tecartus (brexucabtagene autoleucel)	CD19	Mantle cell lymphoma	Kite Pharma/Gilead	FDA approval (2020) [37]
Breyanzi (lisocabtagene maraleucel)	CD19	Large B-cell lymphoma	Bristol-Myers Squibb	FDA approval (2021) [38]
Abecma (idecabtagene vicleucel)	BCMA	Multiple myeloma	Bristol-Myers Squibb	FDA approval (2021) [39]
Carvykti (ciltacabtagene autoleucel)	BCMA	Relapsed or refractory multiple myeloma	Johnson & Johnson/Legend Biotech	FDA approval (2022) [40]

**Table 2:** Some of the most common candidates for optimizing the CAR constructs in terms of domains and protein regions.

Domains	Protein Region
Intracellular	CD3ζ, CD28, and 41BB
Transmembrane	CD3ζ, CD28, 41BB, CD8α, FcγRIIIA, TCRαβ, and DAP12
Hinge domain	CD8α, CD28, IgG4, IgG2, IgG1, and DAP12
Extracellular	VH-VL, VL-VH, and VH

**Table 3:** Some of the key guidelines regarding considerations during the development of CAR T-cell products [64].

Criteria	Description
Eligibility of donor	Donors must be screened for transmissible diseases as per FDA regulations (21 CFR 1271).
Transduction efficiency	Efficiency of gene transduction should be monitored to ensure correct expression of the CAR construct in T cells.
Vector copy number (VCN)	VCN per cell must be monitored and controlled, generally targeting 1–5 copies per cell to avoid toxicity and insertional mutagenesis.
T-cell expansion	Post-transduction, T cells are expanded in a controlled environment, ensuring high viability and potency.
Purity check	The final product must meet specific purity (e.g., CD3+ cells) and identity (e.g., CAR expression) standards.
Potency assays	Potency assays must be performed to ensure CAR T cells are capable of recognizing and killing target cancer cells.
Safety check	Sterility, endotoxin levels, and mycoplasma testing must be performed to ensure no contamination during production.
Viability of CAR T cells	The FDA mandates the minimum viability (usually > 70%) of CAR T cells at the time of infusion.
Cryopreservation	Cells are typically cryopreserved for shipment and storage; freezing methods must ensure cell stability post-thaw. The concentration of DMSO must be ≤10% *v*/*v*; however, ≤5% *v*/*v* is often preferred to further reduce DMSO-related cytotoxicity.
Release criteria	The final product must meet pre-defined release criteria, including CAR expression, viability, identity, purity, and vector copy number (VCN).
Adventitious agent or other contaminants	Testing is required to confirm that the product is free from unintended viruses or contaminants.
Preclinical studies	Preclinical models must demonstrate safety and efficacy before clinical trials can commence.
Clinical manufacturing	Must adhere to Good Manufacturing Practice (GMP) guidelines (21 CFR Parts 210 and 211), including controlled and reproducible processes.
Labeling	CAR T-cell products must have clear labeling for identity, dose, and instructions as per FDA labeling regulations (21 CFR Part 610).
